# Sex differences in vaccine-induced immunity in mice immunized with integrase-defective lentiviral vector delivering the SARS-CoV-2 Spike protein

**DOI:** 10.3389/fimmu.2026.1778067

**Published:** 2026-03-04

**Authors:** Iole Farina, Alessandra Gallinaro, Martina Borghi, Maria Franca Pirillo, Chiara Falce, Andrea Canitano, Zuleika Michelini, Alice Zappitelli, Antonio Di Virgilio, Mario Picozza, Andrea Cara, Donatella Negri

**Affiliations:** 1 Department of Infectious Diseases, Istituto Superiore di Sanità, Rome, Italy; 2 National Center for Global Health, Istituto Superiore di Sanità, Rome, Italy; 3 Center for Animal Research and Welfare, Istituto Superiore di Sanità, Rome, Italy; 4 Core Facilities, Cytometry Area, Istituto Superiore di Sanità, Rome, Italy

**Keywords:** germinal center, integrase-defective lentiviral vector, neutralizing antibody, preclinical study, sex differences, Spike, T cell response, vaccine

## Abstract

**Introduction:**

Integrase-defective lentiviral vector (IDLV) delivering the optimized SARS-CoV-2 Spike protein induces strong and persistent immunity in mice. Here, we investigate potential sex-dependent differences by comparing female and male mice for germinal center (GC) reactions and Spike-specific immune responses induced by IDLV delivering an optimized SARS-CoV-2 Spike Wuhan-Hu-1 protein (IDLV-S).

**Methods:**

Female and male BALB/c mice were injected once intramuscularly with either IDLV-S or IDLV-Mock or were left untreated. Blood, lymph nodes and spleens were collected at selected time points for the analysis of immune responses by flow cytometry, FluoroSpot and neutralization assays.

**Results:**

Strong GC activation was detected at 7 days from the immunization in all vaccinated mice, showing a higher percentage of GC B cells in females. Anti-Spike neutralizing antibodies (nAbs) and T cell responses were detected up to 24 weeks from immunization in all IDLV-S immunized mice. NAbs in sera were more persistent in female than in male mice, and vaccinated females also showed a higher cross-neutralization activity against Spikes from variants of concern, reflecting a better quality of the functional immune response. Both IDLV-S immunized groups showed specific T cell responses evaluated as IFNγ/TNFα producing T cells, with a higher response in females.

**Discussion:**

The higher GC reaction in the immunized females can be the trigger for the more persistent and broader nAb response in females compared to males. Our data confirm sex-dependent vaccine-induced immune responses and support the need of appropriate design of vaccine protocols both at the preclinical and clinical levels.

## 1 Introduction

Sex-based differences in immune responses critically influence the pathogenesis of infectious diseases, vaccine responsiveness and the prevalence of autoimmune disorders. Generally, females display more robust innate and adaptive responses than males (1), exhibiting a lower vulnerability to some microbial infections, but an increased susceptibility to immune-related pathologies (2). Furthermore, heightened immune responses have been observed for a variety of viral vaccines in women, including vaccines against influenza, hepatitis A and B and herpes simplex 2 (3), who nevertheless reported adverse effects more frequently than men following vaccination (4).

These differences are attributed both to genetic and hormonal factors (5, 6), since several immune-related genes are located on the X chromosome and most immune cells express the receptors for sexual hormones, with estrogens able to modulate both innate and adaptive immune responses and to potentiate the immune activation (7, 8). Antibody production is influenced by estrogenic enhancement of somatic hypermutation (9) sex differences in germinal center (GC) formation (10) and in epigenetic accessibility of B cell loci (11). On the contrary, testosterone has a negative effect on T cell proliferation, through the inhibition of IL-2 signaling, and enhances the production of anti-inflammatory cytokines (12). The immunosuppressive effects of testosterone have also been correlated with a reduced neutralizing antibody (nAb) response following influenza vaccination (13).

Studies in mice showed a stronger innate immune response in females compared to males after yellow fever virus or influenza vaccination, due to an upregulation of genes associated with TLR, showing a reduced methylation of TLR7 promoter on X chromosome (14), increased IFN production (4), with enhanced B-cell activation, GC formation (15) and Ig class switch DNA recombination (16).

Although sex-based immunological differences have been discussed in the context of SARS-CoV-2 infection (17, 18), disparities in vaccine-induced immune responses between males and females have not yet been fully addressed. So far, the large studies in men and women evaluated mainly anti-receptor binding domain (RBD) and anti-Spike IgG responses, without measuring nAbs and T cell responses (19). Furthermore, many clinical trials have significant limitations, as they often originate from *post-hoc* analyses, and include different vaccine formulations and immunization protocols (20). Comparing females and males has often not been a priority also in preclinical studies, due to a consolidated sex-bias in experimental research (21, 22). Preclinical mouse models remain good surrogates for human immunity, with advantages lying in the homogeneity of inclusion criteria such as age and diet, the collection of samples from different sites including blood, spleen and draining lymph nodes at different selected time points, and the possibility of using inbred mice, avoiding the need for huge numbers of animals/groups to obtain statistically significant differences.

Here, we aimed at investigating potential sex-dependent differences in the immune responses induced by a SARS-CoV-2 vaccine in the mouse immunogenicity model, by using a Simian Immunodeficiency Virus (SIV)-based Integrase-Defective Lentiviral Vector (IDLV) as delivery platform. We and others previously characterized IDLV as vaccine delivery system in several settings, including viral infectious diseases and tumors in mice (23–25). In addition, IDLV has been tested also in non-human primates, confirming the potency in the induction of strong and persistent immunity (26, 27). The peculiarity of the IDLV-based vaccine is the persistence of the immune responses. The persistence of the antigen expression both at the injection site and in draining LN is directly associated to the strong immunity observed in vaccinated animals (28). We recently developed an IDLV delivering an optimized conformation of SARS-CoV-2 Spike, with modifications that stabilized the Spike protein in the prefusion conformation (29, 30), improving the Spike exposure on IDLV-particles (31), prevented the S1 shedding, and enhanced the exposure of the receptor binding motif (32). This optimized Spike was highly immunogenic in mice after a single immunization, showing increased magnitude of autologous and cross-reactive nAbs compared to the IDLV delivering wild-type Spike and to the subunit vaccine (33).

Therefore, in the present study we took advantage of this efficient vaccine platform to compare functional immune responses in female and male mice vaccinated with IDLV expressing the optimized Spike protein (IDLV-S), focusing on GC activation, anti-Spike nAb kinetics and cross-reactivity, and persistence of Spike-specific T cells.

## 2 Materials and methods

### 2.1 Production of integrase-defective lentiviral vectors for immunization

293T Lenti-X cells (Clontech, Mountain View, CA, USA) were kept in Dulbecco’s modified Eagles medium, with high glucose 4.5 g/L (Gibco, Life Technologies Italia, Monza, Italy) supplemented with 100 units/ml penicillin/streptomycin (Gibco) and 10% fetal bovine serum (Corning, Merk Life Science S.r.l., Milan, Italy), and were utilized for the production of SIV-based IDLV expressing the optimized Spike (IDLV-S-2PFGC, hereinafter referred to as IDLV-S) or the control GFP (IDLV-Mock) by transient transfection, as described (33, 34). In brief, cells (3.5 × 10^6^ cells) were plated on 10 cm Petri dishes (Corning) and transfected with i) the self-inactivating lentiviral transfer vector plasmid pGAE-S2PFGC, encoding the Wuhan-Hu-1 Spike ORF stabilized by the introduction of two consecutive prolines in S2 (2P, K986P and V987P), by functional mutation of RRAR into GSAS in the furin cleavage site (FCS), by inclusion of the D614G mutation and by deletion of the terminal 21 amino acid (aa) of the cytoplasmic tail (delta21) (33) or pGAE-GFP, encoding the control antigen GFP, ii) the SIV-based integrase-defective packaging plasmid pAdSIVD64V (33, 35) and iii) the phCMV-VSV.G plasmid, expressing the pseudotyping vesicular stomatitis virus envelope glycoprotein G (VSV.G) (33, 35), using JetPrime transfection kit (Polyplus Transfection, Illkirch, France) following the procedure of the manufacture. Forty-eight hours post transfections, the cell culture supernatants containing the vectors were harvested, filtered through a 0.45 μm pore size filter (Millipore Corporation, Billerica, MA, USA) and ultracentrifuged at 65,000 × g at 4°C for 2.5 h on a 20% sucrose cushion. Vector particles were resuspended in 1× phosphate buffered saline (PBS, Gibco) and stored at −80 °C. Each stock of IDLV was titered using the reverse transcriptase (RT) activity assay and the corresponding transducing units (TU) were determined by comparing the recovered RT activity with that of IDLV-GFP virions with known infectious titers (36).

### 2.2 Mouse immunization protocol

Female and male BALB/c mice were acquired from Envigo RMS (San Pietro al Natisone, Udine, Italy) and subsequently kept in the animal facility at the Istituto Superiore di Sanità (ISS, Rome, Italy) under pathogen-free conditions. Procedures involving all murine protocols were always performed in accordance with the Italian legislation and European Union guidelines for animal care. Studies were authorized by the Italian Ministry of Health (Authorization n. 731/2020-PR, 21/7/20, prot. D9997.107) and were reviewed by the Service for Animal Welfare at ISS. Seven weeks old female and male mice were immunized once intramuscularly with 10 × 10^6^ RT units/mice of IDLV-S or IDLV-Mock or left untreated. Sampling of retro orbital blood was performed with glass Pasteur pipettes and sera were collected prior the immunization and monthly thereafter and stored at -80 °C. One week or 24 weeks after immunization, mice were euthanized by cervical dislocation and draining lymph nodes (LNs) and spleens were collected and processed for the analysis of cellular responses, including quantification of germinal center (GC) B cells and T follicular helper (Tfh) cells by flow cytometry, and Spike-specific T cells by FluoroSpot.

### 2.3 Flow cytometry

To evaluate the GC activation in the IDLV-S immunized mice, draining inguinal and popliteal LNs were collected 1 week after the immunization and homogenized into a single-cell suspension. Cells were stained with live/dead near IR (Thermo Fisher Scientific, Waltham, MA, USA) and Fc blocked with anti-CD16/CD32 monoclonal antibody (cat. number 156604, BioLegend, San Diego, CA, USA, 1:200) prior to staining. The cells were afterward stained with a mixture of monoclonal antibodies, including anti-CD19 Brilliant Violet 421™ (cat. number 152421, 1:500), anti-GL7 PE (cat. number 144608, 1:200), anti-Fas AF647 (cat. number 152620, 1:500), anti-CD4 FITC (cat. number 100510, 1:500), anti-PD-1 Brilliant Violet 421™ (cat. number 135217, 1:200), anti-CXCR5 PE/Cyanine7 (cat. Number 145516, 1:50), which were purchased from BioLegend and anti-CD3 BB700 (cat. Number 745836, 1:200) purchased from BD Bioscences (Franklin Lakes, NJ, USA). Next, the cells were fixed, resuspended in PBS, and analyzed on a Cytoflex S flow cytometer (Beckman Coulter Life Sciences, Brea, CA, USA), using CytExpert analysis software (Beckman Coulter Life Sciences). Gating strategies are showed in **Supplementary Figure 1**.

### 2.4 Pseudovirus titration and neutralization assay

Lentiviral vectors expressing luciferase (LV-Luc) and pseudotyped with Spike variants were generated by transient transfection of 293T Lenti-X cells, using pGAE-LucW transfer vector expressing luciferase, the integrase competent packaging plasmid pAdSIV3+, and each pseudotyping plasmid expressing the Spike proteins from SARS-CoV-2 VoC, as previously described (33, 37). In brief, plasmids pSpike-C3, pSpike-INC3, pSpike-BA.1C3, pSpike-BA.2C3 and pSpike-BA.4/5C3, encoding respectively the codon optimized cytoplasmic-truncated Spike ORF from Wuhan-Hu-1, B.1.617.2 (Delta), B.1.1.529 (Omicron) BA.1, BA.2 and BA.4/5 were used for pseudotyping the LV-Luc. All preparations of LV-Luc pseudoviruses were titered on Vero E6 cells (Cercopithecus aethiops derived epithelial kidney, ATCC C1008) as previously described (37).

For neutralization assay, serial 2-fold dilutions of sera starting from 1:80 were incubated in duplicate with each LV-Luc pseudotype (3 × 10^5^ relative light units, RLU) at 37 °C for 30 min in 96-deep well plates (Resnova, Roma, Italy). The mixture was then added to Vero E6 cells (2.2 × 10^4^ cells/well) seeded in a 96-well Isoplate (Revvity, Inc, Waltham, MA, USA). Cell-only and virus-only controls were included. After forty-eight hours, luciferase expression was measured by using the Britelite plus Reporter Gene Assay System (Revvity, Inc.). The inhibitory dilution (ID) 50 reported in the results corresponds to the dilution of serum showing 50% inhibition of the infection (corresponding to neutralization), calculated from virus-only control wells (100% infection). ID50 was calculated using a linear interpolation method (31).

### 2.5 IFNγ/TNFα FluoroSpot assay

Suspensions of splenocytes were obtained following mechanical disruption of spleens in the presence of 3 mL of Ammonium-Chloride-Potassium (ACK) lysing buffer followed by passage through cell strainers (Corning). Splenocytes were then washed using complete medium (RPMI 1640 medium supplemented with 1% penicillin/streptomycin, 2 mM L-glutamine, 10% FBS, and 50 mM 2-mercaptoethanol), centrifuged at 1500 rpm for 10 min at 4 °C, and then counted. The assay was performed using Mabtech reagents and protocol (FluoroSpot Plus, Mabtech AB, Sweden). Splenocytes were seeded in 96-well plates, previously coated with anti-IFNγ and anti-TNFα antibodies, at a density of 2.5 × 10^5^/well and thereafter stimulated overnight either with 1 µg/mL of SARS-CoV-2 Wuhan Spike peptide pool of 15-mer sequences with 11 amino acids overlap (PepTivator^®^ SARS-CoV-2 Prot_S complete, Miltenyi Biotec, Bologna, Italy) or with 5 µg/ml of a H2-D^d^-restricted SARS-CoV-2-Spike 9-mer peptide (KNKCVNFNF, ProImmune Ltd., Oxford, UK (38, 39), as specific stimulation. Positive control included Concanavalin A (5 µg/mL, Sigma Chemicals; St. Louis, MO, USA), while the complete medium was used as negative control. Wells were washed and incubated with anti-IFNγ and anti-TNFα detection antibodies and then with fluorophore-conjugates for 60 min. The number of Spot Forming Cells (SFC) was counted using a FluoroSpot reader (AID iSpot, AID GmbH, Strassberg, Germany) and results were expressed as SFC/10^6^ cells. The number of SFC counted in the wells treated with the medium (background) was subtracted from the number of SFC counted in the wells treated with the specific peptides. Samples were recorded positive when the number was equivalent to at least 50 specific SFC/10^6^ cells and was at least two-fold higher than the background values.

### 2.6 Statistical analysis

Data and graphs were prepared using GraphPad Prism 9.4.1 (GraphPad Software Inc., San Diego CA, USA). The normality of data distributions was verified by the Shapiro-Wilk test. Data were analyzed by repeated-measure (RM) one-way ANOVA or unpaired t test, as indicated. Values of P < 0.05 were considered to infer statistical significance.

## 3 Results

### 3.1 IDLV-S induces strong GC activation in immunized mice

**Figure 1:**
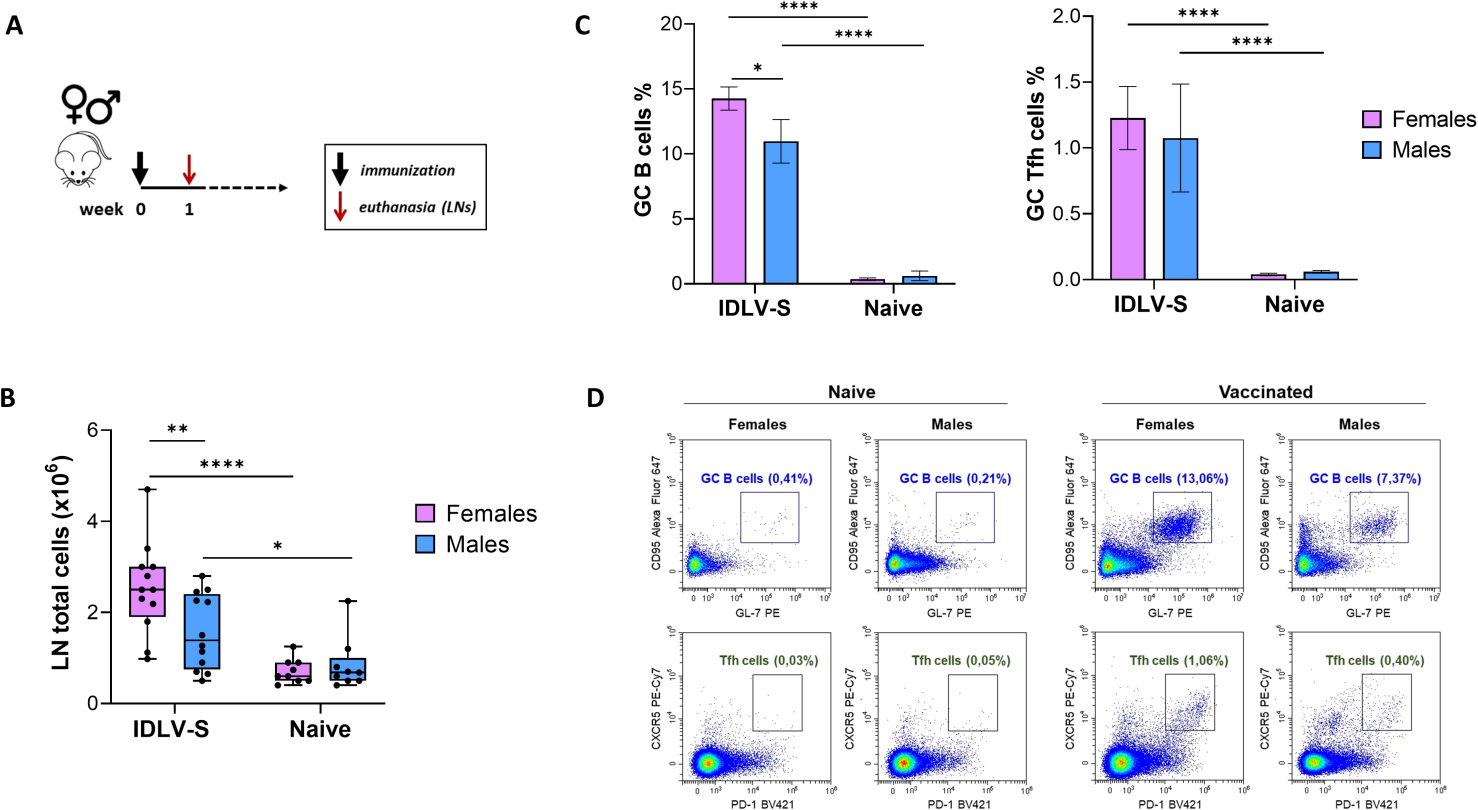
Analysis of GC activation in IDLV-S vaccinated mice. **(A)** Schedule of immunization. Female and male BALB/c mice were immunized with IDLV-S (12 females and 12 males) or left untreated (naïve, 9 females and 9 males) and euthanized 1 week after vaccination. Draining LNs were collected for the analysis of the GC response. **(B)** Cells isolated from draining LNs were counted using trypan blue. Data are expressed as total live cells. Box whiskers are shown, with each dot representing one animal. Asterisks indicate a statistically significant difference between groups (unpaired t test, *p<0.05, **p<0.01, ****p<0.0001). **(C)** Comparison of GC B cells (left) and Tfh cells (right) between females and males. Data are expressed as percentages of GC B cells on total B cells and percentage of GC Tfh cells on total CD4+ cells (mean ± SEM). Asterisks indicate a statistically significant difference between groups (unpaired t test, *p<0.05, ****p<0.0001). **(D)** Identification of GC B cells and Tfh cells by flow cytometry. Representative dot plots show GC B cells (CD3- CD19+ GL7+ CD95+, upper panels) and GC Tfh cells (CD3+ CD4+ CXCR5+ PD1+, lower panels) from naïve and from vaccinated females vs males.

We first investigated the GC responses after IDLV vaccine injection in both female and male mice. Groups of female and male BALB/c mice were immunized intramuscularly with IDLV-S (N = 12 per sex) or left untreated (naïve, N = 9 per sex). The draining inguinal and popliteal lymph nodes (LNs) were collected 1 week after the immunization (**Figure 1A**). The total number of the LN derived lymphocytes was significantly higher in vaccinated mice compared to naïve, as expected (p<0.0001 and p<0.05 in females and males, respectively), as well as in females compared to male mice (p<0.01) (**Figure 1B**). To detect GC B cells and Tfh cells, lymphocytes were stained and analyzed by flow cytometry. GC B cells were evaluated as CD3- CD19+ GL7+ CD95+ and the Tfh cells as CD3+ CD4+ CXCR5+ PD1 +. The gating strategy for the identification of the indicated cell populations is depicted in **Supplementary Figure 1**. One week after immunization percentages of GC B cells and Tfh cells were significantly increased in the IDLV-S vaccinated groups compared to naïve mice, irrespective of sex (**Figure 1C**). In particular, GC B cells ranged from 7.4 to 16.3% of total CD19+ cells and the Tfh cells ranged from 0.4 to 2.4% of the CD4+ T cells, while the percentage of GC B cells and of Tfh cells in naïve mice were much lower, ranging between 0.25-0.99 and 0.03-0.05, respectively. Importantly, vaccinated females showed significantly higher GC B cells compared to males (p<0.05). Tfh cells were higher in females, but a large variability observed in both sexes probably hindered statistical significance (**Figure 1C**). Flow cytometry analysis from a representative experiment is shown in **Figure 1D**. Overall, IDLV-S immunization elicits a robust GC response characterized by coordinated GC B-cell and Tfh-cell activation, with a preferential expansion of GC B cells in females, suggesting that sex-dependent differences in early GC dynamics may contribute to the magnitude and durability of humoral immunity.

### 3.2 Level, kinetics and cross-neutralization of anti-Spike neutralizing antibodies are sex-dependent in IDLV-vaccinated mice

**Figure 2:**
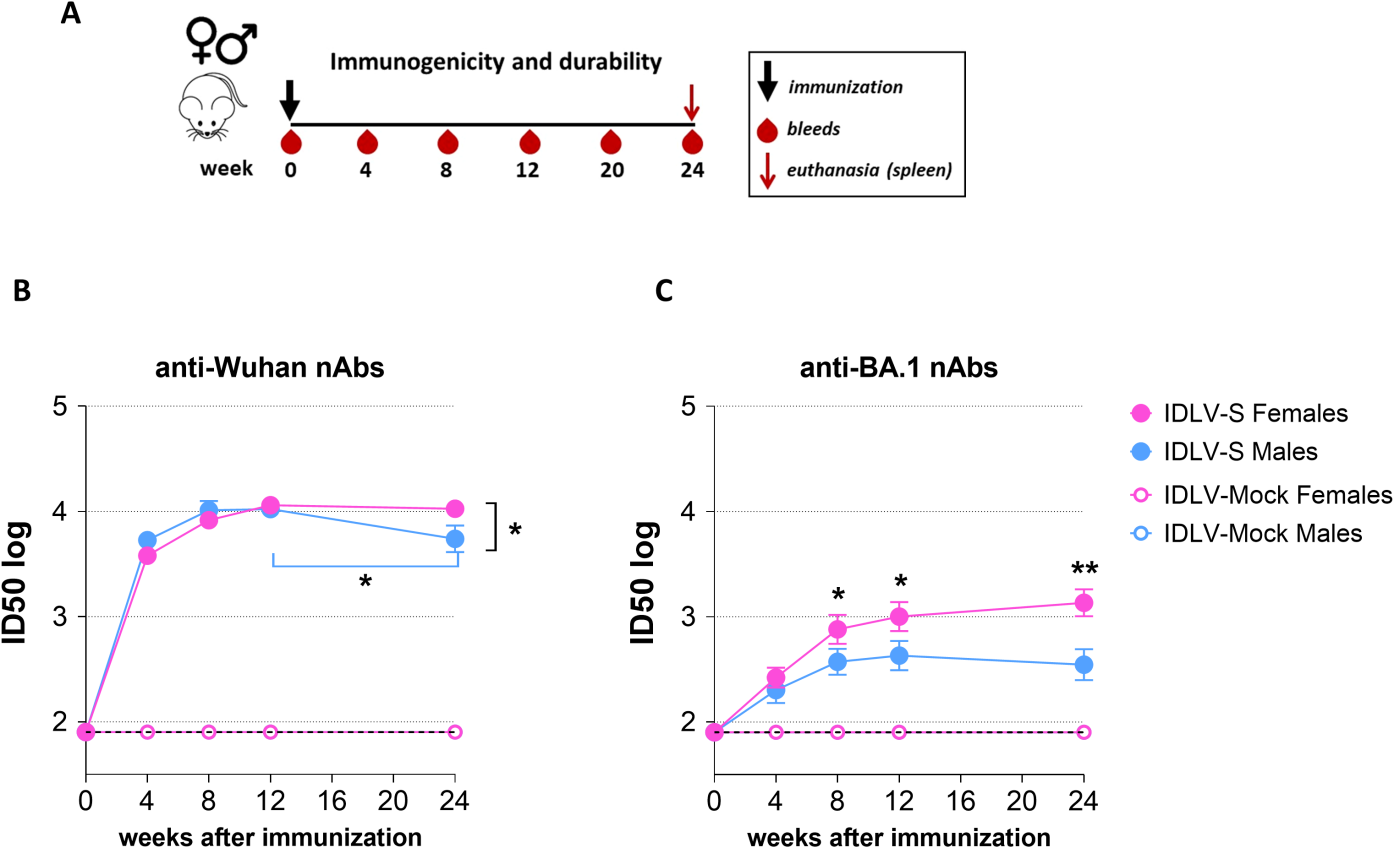
Sex difference in IDLV-S-induced anti-Spike neutralizing antibodies (nAbs). **(A)** Schedule of immunization and sampling. Female and male BALB/c mice were intramuscularly immunized with IDLV-S (15 females and 14 males) or IDLV-Mock (4 females and 4 males) and euthanized 24 weeks after the immunization. Serum was collected at the indicated time points after immunization. **(B)** Kinetics of anti-Spike Wuhan-Hu-1 nAbs and **(C)** anti-Spike Omicron BA.1 nAbs in sera. Results are expressed as mean ID50 ± SEM. The dotted lines indicate the assay cut off (minimum serum dilution tested 1:80 dilution). Asterisks indicate a statistically significant difference between groups (unpaired t test, *p<0.05, **p<0.01).

To test the immunogenicity of IDLV-S and the durability of the vaccine induced responses, groups of female or male mice were vaccinated once with IDLV-S or IDLV-Mock and the Spike-specific immune response was monitored for up to 6 months (**Figure 2A**).

The kinetics of nAbs in serum of immunized mice were evaluated with a pseudovirus assay based on lentiviral vector expressing luciferase (LV-Luc) pseudotyped with Spike derived from Wuhan-Hu-1 and VoC, as previously described (31, 33, 37, 40, 41). IDLV-S vaccine induced high levels of nAbs against the Wuhan-Hu-1 Spike, homologous to the vaccine strain (**Figure 2B**). In females the level of nAbs peaked at 3 months and remained stable up to 6 months, while immunized males showed a peak at 3 months which significantly decreased thereafter (p<0.01 between 12 and 24 weeks within males), determining a significant difference of nAb levels between females and males 24 weeks post-immunization (p<0.05). The difference was more pronounced when we analyzed the kinetics of nAbs against the Omicron BA.1 Spike protein. As shown in **Figures 2C** the levels of heterologous nAbs were higher in females than in males in almost all the assayed time points (p<0.05; p<0.01). No specific anti-Spike neutralizing activity was detected in the IDLV-Mock immunized mice, as expected.

**Figure 3:**
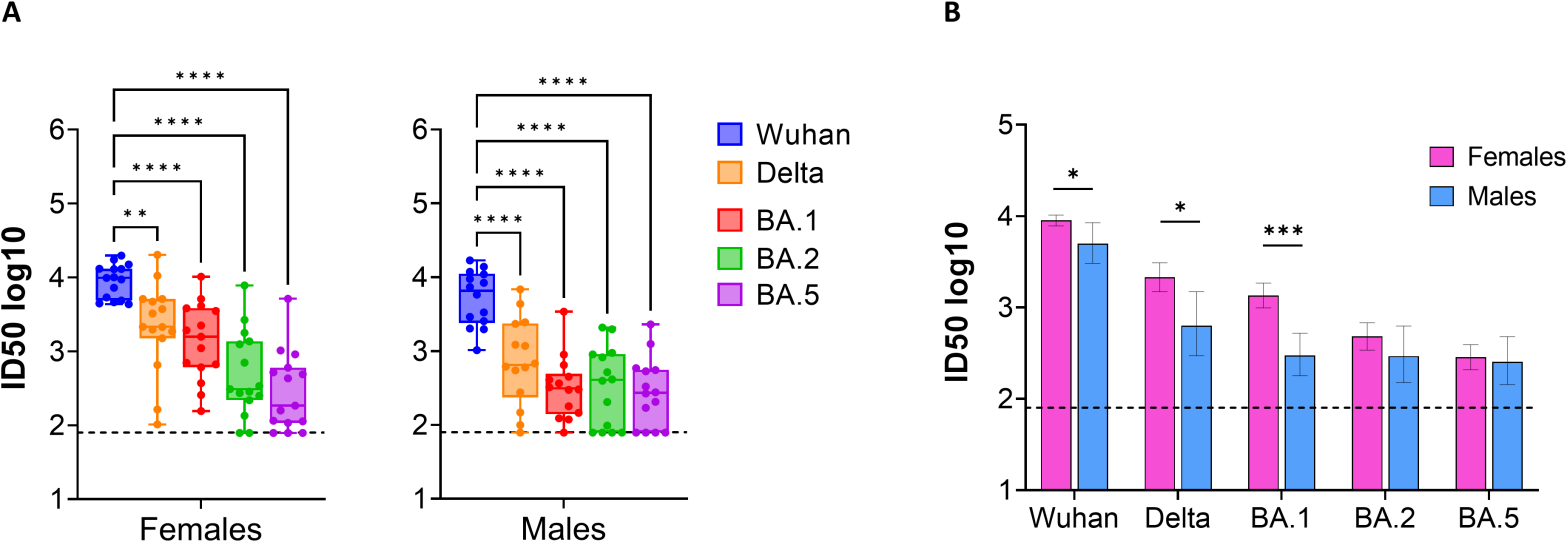
Cross-neutralization activity elicited by IDLV-S immunization. **(A)** NAbs against each VoC were measured in serum samples collected at 20 weeks after immunization, using pseudoviruses carrying the Spike protein from the indicated VoC. Results are expressed as log10 ID50. Box whiskers are shown, with each dot representing a single mouse. Asterisks indicate a statistically significant difference between groups (RM one-way ANOVA, **p<0.01, ****p<0.0001). **(B)** Comparison between females and males. Data are expressed as mean ID50 ± SEM. Asterisks indicate a statistically significant difference between groups (unpaired t test, *p<0.05, ***p<0.001). The dotted lines indicate the assay cut off (minimum serum dilution tested 1:80 dilution).

Serum samples from all IDLV-S vaccinated mice collected at 20 weeks post-immunization were assayed for cross-neutralization activity against the Spike protein from Delta and Omicron BA.1, BA.2 and BA.4/5 VoC. As shown in **Figure 3A**, nAb titers against VoC were significantly lower compared to those measured against Wuhan-Hu-1 Spike, homologous to the vaccine sequence, and ID50 values were Delta > Omicron BA.1 > BA.2 > BA.4/5, showing a similar pattern in both sexes. However, we observed higher cross-neutralizing titers against VoC in females, with a statistically significant difference between sexes for anti-Delta and anti-BA.1 nAb titers (p<0.05 and p<0.001, respectively) (**Figure 3B**). Overall, these data demonstrate that a single IDLV-S immunization elicits durable anti-Spike nAb responses with distinct sex-dependent features.

### 3.3 Persistence of IDLV-S induced T cell immunity is higher in females

**Figure 4:**
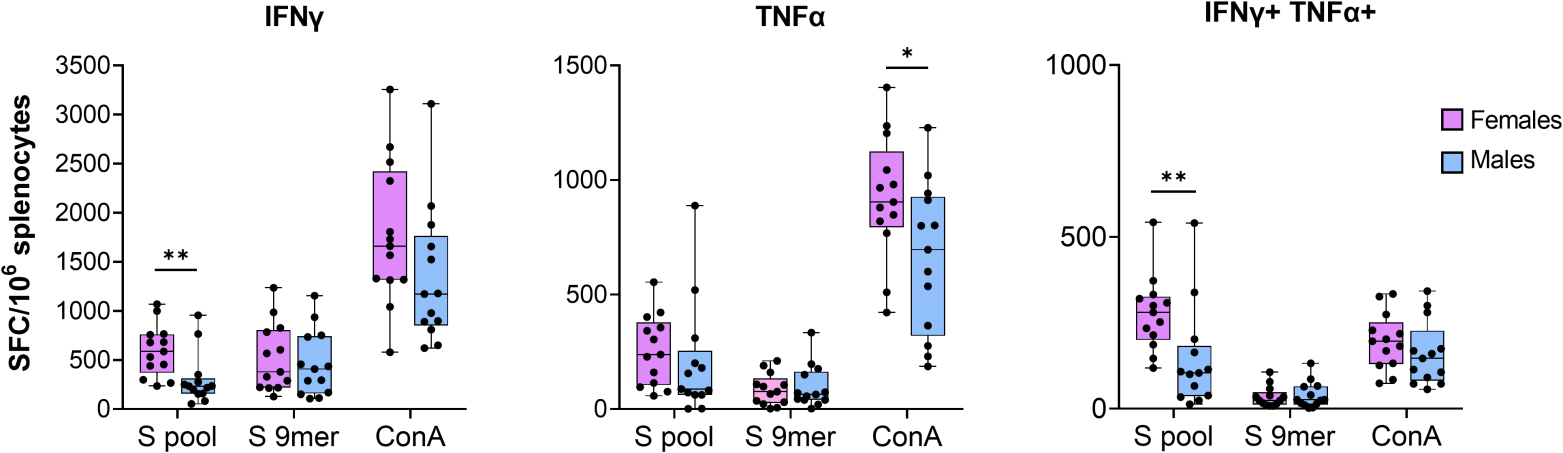
Anti-Spike specific T cell immunity. Splenocytes recovered 24 weeks after immunization with IDLV-S were assayed for IFNγ and TNFα producing T cells by IFNγ/TNFα FluoroSpot. Cells were stimulated overnight with Spike peptide pool or the MHC-I-restricted peptide. Single and double cytokine producing T cells are shown. Data are expressed as mean (± SEM) specific Spot Forming Cells (SFC) per million splenocytes. Each dot represents a single mouse. Asterisks indicate a statistically significant difference between groups (unpaired t test, *p<0.05, **p<0.01).

IFNγ/TNFα FluoroSpot was performed on splenocytes 24 weeks after immunization using a pool of 15-mer peptides covering the entire Wuhan Spike protein and an immunodominant MHC-I-restricted Spike-derived peptide (38, 39). As shown in **Figure 4**, Spike-specific IFNγ, TNFα and double positive T cells were induced in all mice vaccinated with IDLV-S. In particular, the response was higher in females, as shown by the number of cytokine-producing T cells upon stimulation with ConA and Spike pool, whereas the stimulation with MHC-I-restricted peptide did not induce a statistically significant different response between females and males. Mice immunized with IDLV-Mock did not show any Spike-specific T cell response and ConA stimulation induced higher, but not significant, number of SFC in females (data not shown). These findings confirm that IDLV-S vaccination induces a durable Spike-specific T cell immunity which is detectable at late time points, with females displaying a higher magnitude of multifunctional cytokine-producing T cell responses.

## 4 Discussion

In this study, we investigated potential sex-based differences in vaccine-induced anti-SARS-CoV-2 immune responses in mice by using a SIV-based IDLV expressing an optimized Spike protein. Although sex-related differences in safety and efficacy of several vaccines have been largely discussed, few studies analyzed the sex influence on COVID-19 vaccine functional immune responses, obtaining controversial results due to unbalanced sex representation in clinical trials, heterogeneity in methods to measure vaccine outcomes and different inclusion parameters (42).

It is now widely acknowledged that anti-Spike nAbs represent the primary correlate of protection from COVID-19 (43–45) and that potency of antibody response to vaccination generally depends on GC activation (46–48). GCs are micro anatomical structures that form within lymphoid tissues following exposure to antigens and where somatic hypermutation and affinity maturation occur. Therefore, we analyzed the GC reaction after immunization with IDLV by measuring activated GC B cells and Tfh cells in females and males. We observed a strong reaction of GC in IDLV-S immunized mice compared to naïve mice in terms of both increased number of total lymphocytes and higher percentage of the analyzed GC subpopulations. Importantly, we detected a marked increase in the percentage of GC B cells in female compared to male mice. Since B cells compete for antigen and mutate their B cell receptors within GC, the higher GC B cells percentage found in females could explain the better B cell immunity detected in IDLV-S vaccinated females, in terms of levels and quality of nAbs. Indeed, previous reports demonstrated that anti-SARS-CoV-2 mRNA vaccines promote the GC B cells response, which strongly correlate with nAb production in both mice and humans (49–51). In addition to the Spike immunogen delivered by IDLV both as transgene and pseudotyping protein, part of the GC response can be attributable to the delivery system *per se.* However, based on the strong and persistent Spike specific immunity induced by IDLV-S immunization in terms of both nAbs and T cell responses, we expect that a proportion of GC B and T cells are Spike-specific.

Although females and males showed a similar induction of autologous nAbs, the magnitude of the response remained stable overtime in females, achieving significantly higher titers six months after the IDLV-S administration. Indeed, in males autologous nAb levels significantly decreased at 24 weeks post-immunization compared to the peak response observed at 12 weeks. Considering that affinity maturation in GC leads to the generation of long-lived plasma cells, which in turn provide a sustained antibody-mediated immunity (52), we hypothesize that the greater GC reaction observed in IDLV-S vaccinated females triggered the more persistent nAb response detected in females compared to males. Sex differences were more evident when we evaluated the ability of IDLV-S to induce cross-reactive nAbs against Spikes from SARS-CoV-2 VoC. Both groups exhibited a similar trend, with anti-Delta nAb levels being higher than those against the Omicron VoC, which was expected and possibly due to the greater genetic distance from the ancestral strain, autologous to the vaccine, as previously described (33). However, enhanced cross-reactivity was evident in females. In particular, anti-BA.1 nAbs were always higher in females during the entire period of analysis. These results are consistent with a previous report showing that, following influenza vaccination, female mice exhibited higher cross-protection against challenging mutant viruses compared to males, due to increased production of class-switched, somatically hypermutated antibodies in female GC B cells (53). In this context, the higher GC reaction in the IDLV-S immunized females can be the trigger for the more persistent and broader nAb response in females compared to males.

In this study, we confirmed that IDLV-S elicited specific and sustained anti-Spike cellular response in mice, as we previously reported (33). In addition, here, we investigated the differences in IDLV-S-induced T cells immunity between female and male mice. All IDLV-S vaccinated mice showed specific and durable T cell immunity, with IFNγ and TNFα levels present at six months post-immunization in both females and males while were undetectable in mice vaccinated with IDLV-Mock. Regarding sex disparities, we observed an overall higher T cell activation in females following stimulation with the Spike peptide pool, confirming literature data showing a better adaptive T cell response in females both in preclinical and clinical settings (3). Considering that T cells are involved in limiting the viral replication and circulation to distal organs (54) and that most of T cells epitopes are conserved among VoC (55, 56), the evaluation of long-lasting cellular responses should be considered in the context of sex differences. Of note, we did not observe significant differences between sexes when the Spike specific MHC-I restricted peptide was used for stimulation of splenocytes, suggesting that sex-dependent modulation of T cell immunity may be more apparent at the level of epitope breadth and clonal diversity, which is better reflected by peptide pool stimulation than by a single MHC-I–restricted peptide. Alternatively, sex-related differences may preferentially influence CD4^+^ T cell help and cytokine production rather than CD8^+^ T cell responses to individual epitopes.

The link of TLR7 to the X chromosome could be partly responsible for the higher immune response observed in females in our study. It has been shown that *in vivo* injection of LV induces a transient activation of innate response by interaction with TLR3 and TLR7 (57). The TLR7 gene often escapes X-inactivation, resulting in higher expression levels of the receptor in female plasmacytoid dendritic cells (pDC) and B cells, leading to higher production of type I IFNs and pro-inflammatory cytokines upon activation and promoting B-cell activation and GC responses, particularly in response to viral infections (58, 59). It is important to note that lentiviral vectors pseudotyped with VSV.G, as the IDLV used in our vaccine studies, efficiently transduces DC (60, 61), while is unable to transduce B cells (62). In our previous studies evaluating the inflammatory and innate response after IDLV injection in mice, we observed that IDLV induced a mild inflammation at the site of injection and a mild innate response (28, 63). These data were confirmed by other groups showing very weak pro-inflammatory properties of lentiviral vectors (23). In particular, although the immune response in mice immunized with lentiviral vectors is partly dependent on TLR7 signaling, as demonstrated by the weaker cellular immunity observed in TLR7 KO vaccinated mice (64), the overall *in vivo* activation of pDC is modest and there are other pathways than TLR7 signaling used by IDLV *in vivo* to elicit immune responses (63).

This study has limitations. In particular, we evaluated the sex differences after IDLV vaccination only in BALB/c mice and we did not perform challenge experiments. To fully understand the mechanisms underlying the sex differences in the vaccine effectiveness, efficacy studies including the SARS-CoV-2 challenge should be performed. Also, comparison with other relevant vaccine strategies, such as mRNA and adjuvanted protein, will be necessary to dissect the peculiarity of each delivery system. Additional factors, including the innate immune response, hormonal status and the analysis of Spike-specific GC B cells and Tfh cells, should be further investigated in order to achieve a more comprehensive understanding of sex disparities in immunogenicity following IDLV vaccination.

Overall, our data confirmed the remarkable utility of IDLV as a versatile platform for the delivery of prototypic vaccines in preclinical studies, due to its ability to elicit GC reaction and cross-reactive and persistent humoral and cellular immunity. We showed that sex differences might influence the IDLV-induced immune response, suggesting to consider sex dimorphism in the design and selection of new vaccine candidates and in the optimization of vaccine regimens.

## Data Availability

The raw data supporting the conclusions of this article will be made available by the authors, without undue reservation.
