# Supplementary figures and images for: Sex differences in vaccine-induced immunity in mice immunized with integrase-defective lentiviral vector delivering the SARS-CoV-2 Spike protein
Source: Front Immunol. 2026 Mar 4;17:1778067. doi: 10.3389/fimmu.2026.1778067 (PMC12996097; doi:10.3389/fimmu.2026.1778067)

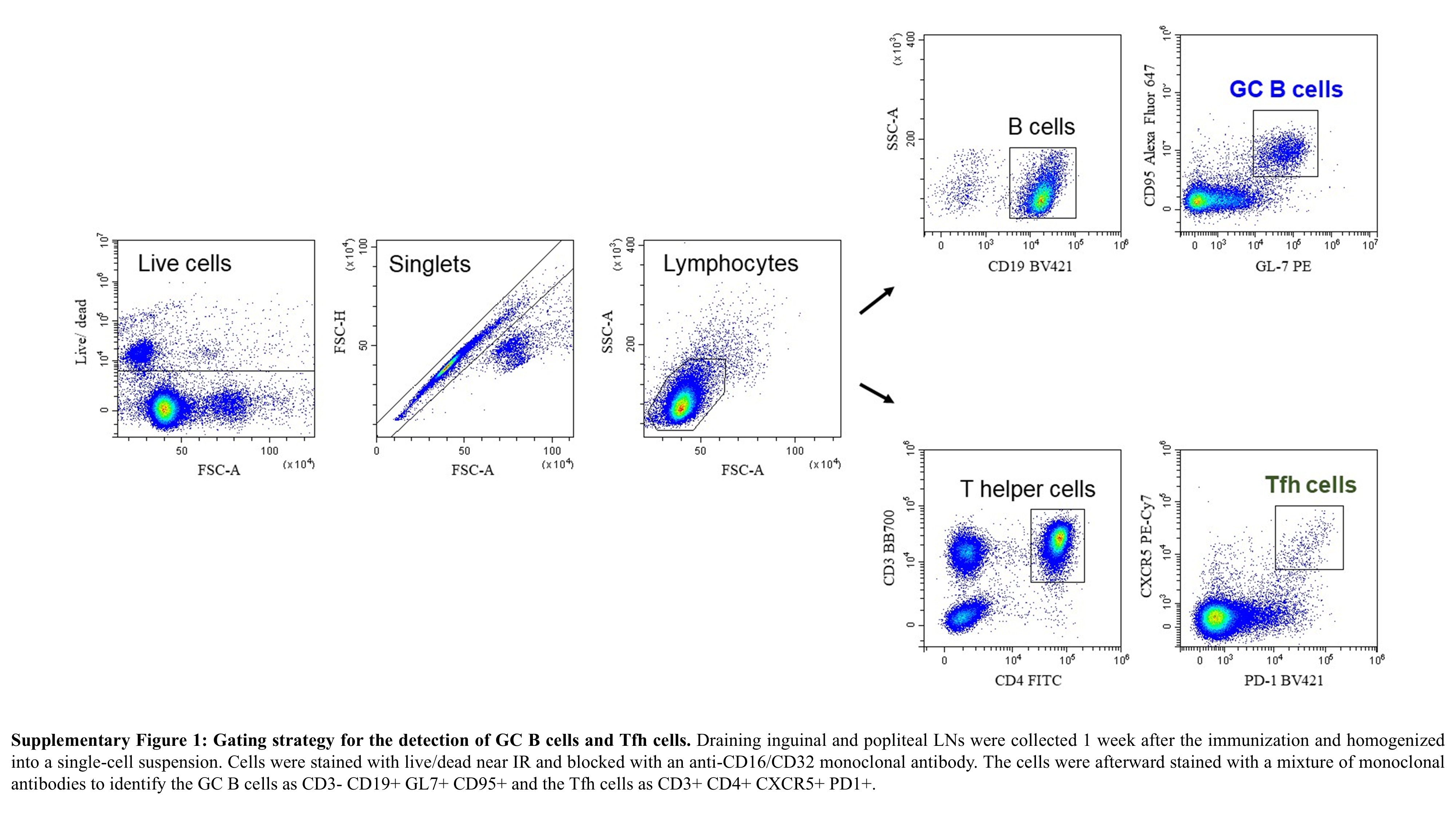

Supplement: Supplementary file 1 [file Image1.tif]
